# Mesoscopic Segregation of Excitation and Inhibition in a Brain Network Model

**DOI:** 10.1371/journal.pcbi.1004007

**Published:** 2015-02-11

**Authors:** Daniel Malagarriga, Alessandro E. P. Villa, Jordi Garcia-Ojalvo, Antonio J. Pons

**Affiliations:** 1 Departament de Física i Enginyeria Nuclear, Universitat Politècnica de Catalunya, Terrassa, Spain; 2 Neuroheuristic Research Group, Faculty of Business and Economics, University of Lausanne, Lausanne, Switzerland; 3 Department of Experimental and Health Sciences, Universitat Pompeu Fabra, Barcelona, Spain; Hamburg University, GERMANY

## Abstract

Neurons in the brain are known to operate under a careful balance of excitation and inhibition, which maintains neural microcircuits within the proper operational range. How this balance is played out at the mesoscopic level of neuronal populations is, however, less clear. In order to address this issue, here we use a coupled neural mass model to study computationally the dynamics of a network of cortical macrocolumns operating in a partially synchronized, irregular regime. The topology of the network is heterogeneous, with a few of the nodes acting as connector hubs while the rest are relatively poorly connected. Our results show that in this type of mesoscopic network excitation and inhibition spontaneously segregate, with some columns acting mainly in an excitatory manner while some others have predominantly an inhibitory effect on their neighbors. We characterize the conditions under which this segregation arises, and relate the character of the different columns with their topological role within the network. In particular, we show that the connector hubs are preferentially inhibitory, the more so the larger the node's connectivity. These results suggest a potential mesoscale organization of the excitation-inhibition balance in brain networks.

## Author Summary

One of the salient characteristics of neurons is their ability to either excite or inhibit other neurons, depending on the type of neurotransmitter they use to act upon them. In fact, a careful balance between excitation and inhibition is required for the brain to operate in a sustained state, away from both epileptic activity (which would arise if excitation dominated over inhibition) and complete quiescence (which would emerge if inhibition prevailed). While much work has been devoted to study how excitation and inhibition are organized at the level of neural microcircuits, little is known about how these two effects are structured at larger scales. Here we are interested in the scale of cortical macrocolumns, large collectives of neurons that can be described computationally by population models known as neural mass models. These models are routinely used to describe the brain rhythms observed with non-invasive techniques such as EEG. In this paper we show, using a neural mass model, that a collection of coupled cortical macrocolumns self-organizes spontaneously into a dynamical state in which excitation and inhibition are segregated at the mesoscopic level, with some cortical columns (the most connected ones) being inhibitory, and the rest being excitatory.

## Introduction

The correct operation of the brain requires a high degree of self-organization, one of whose paradigmatic examples is a careful balance between excitation and inhibition at the neuronal level [1–3]. Much effort has been devoted to understand the role played by the excitatory/inhibitory balance (understood as an equilibrium either among neurons within a network, or along time in a given neuron) to control the neuronal dynamics and to achieve regular and irregular synchronization at the cellular level [4–11]. The presynaptic irregularities generate extremely complex and nontrivial effects on postsynaptic neurons through inhibitory synapses even in the presence of constant inputs [12–14]. Functionally, control in the excitation/inhibition balance is known to underlie a wide range of phenomena including sensory adaptation [15, 16], slow-wave sleep [17], signal tuning [18, 19], motor control [20], and sound localization [21], among many other behaviors.

The columnar structure of the cerebral cortex was initially described by Lorente de Nó [22] as a morphological unit, usually referred to as “minicolumn”, formed by several tens of neurons [23–25]. The existence of a strong interaction between several tens of minicolumns into a larger functional unit was initially recognized in the motor system [26], extended to the entire neocortex [27] and referred to as “hypercolumn” in the visual cortex [28] and “macro column” in general [29]. The mesoscopic neuronal populations belonging to cortical macrocolumns composed of thousands of neurons exhibit coherent dynamical responses to sensory [30] and thalamic [31] stimuli. Sensory stimulation has also been seen to lead to coherent oscillations between neighboring macrocolumns [32]. These observations indicate that brain dynamics can be studied (at least partly) at a mesoscopic scale, and it is thus worth asking whether the excitation/inhibition balance is somehow structured at this level as well [33]. This is the question that we address in this paper.

In order to describe the dynamics of cortical macrocolumns we use a neural mass model (NMM) originally developed by Jansen and co-workers to reproduce the evoked EEG activity elicited in neuronal populations by visual stimuli [34, 35], in terms of their average postsynaptic potential [36]. Since their introduction [37], neural mass models have been widely used, in both the temporal [38] and spectral domains [39], to explore a broad range of phenomena that includes spontaneous activity in the visual cortex [40], local generation of multiple rhythms [41], rhythm propagation across cortical areas [42], inter-laminar dynamics and plasticity [43], generation [44] and termination [45, 46] of self-organized transients in epileptogenic tissue, and critical dynamics near instability regions [47], among many other phenomena. Due to their versatility and relevance for brain modeling, neural mass models have become one of the main levels of description in computational modeling environments such as the Virtual Brain simulator [48].

Neural mass models have a rich bifurcation structure [49] that is substantially augmented by coupling. Two coupled NMMs, for instance, readily lead to chaotic dynamics [50]. Graph theoretical analysis of networks of coupled NMMs have been used to investigate the relationship between structural and functional connectivity [51]. In the same spirit, studying the dynamics of realistic full-brain networks of NMMs [52] allows to investigate the relationship between the anomalous connectivity at the structural and functional levels in cognitive disorders [53]. But the question still remains as to how the detailed topological properties of mesoscopic brain networks determine the dynamical behavior of neural tissue, in particular with respect to the balance between excitation and inhibition.

In order to explore the organization of excitation and inhibition in brain networks, we have to make assumptions regarding the architecture of those networks. The nature of the topology of brain networks has been under intense investigation and debate in recent years [54–59]. Functional brain networks have been shown to display both small-world [60, 61] and scale-free [62–67] features. From the structural point of the view, on the other hand, several studies have reported the existence of inhomogeneous connectivity features dominated by hubs [68–72], which is consistent with findings indicating an increased sensitivity of cortical networks to localized lesions acting upon specific nodes [73]. Accordingly, in what follows we assume a power-law (scale-free) distribution of network connectivity, and study how this connectivity profile affects the excitation/inhibition dynamics of brain networks at the mesoscopic level. The results shown below, however, are not specific to the connectivity profile assumed, since they also hold for both scale-free networks with different clustering (see Supporting Information Fig. S1) and other network topologies such as small-world or random networks (see Supporting Information Fig. S2).

## Results

We focus in networks formed by 50 identical nodes, whose dynamics is represented by an extension of the model by Jansen and co-workers [34, 35]. Each node is meant to represent a cortical macrocolumn, and we consider periodic driving mimicking sensory input through the thalamus [74]. Before addressing the dynamical behavior of the complete network, we characterize the dynamics of a single column when varying the amplitude and the frequency of its driving. In these conditions different responses of the driven isolated node (periodic, quasi-periodic or chaotic) are obtained [74], as described in what follows.

### Single node activity

The activity of an isolated cortical column, corresponding to a node in our network, is represented by a neural mass model, as described in the Materials and Methods section (see Eqs. 1–11)). For an external stimulation level (given in our model by the input pulse density $\bar{p}$) within a biologically plausible range and the parameters used here (see Table 1), this model is known to lead to limit cycle oscillations [49, 75] with a frequency of around 10.8 *Hz*, corresponding to the alpha band.

**Table 1:** Model parameters. All parameters have been adapted from Jansen et al. [35] except for the sinusoidal input in Eq. (6).

**Parameter**	**Value**
Cortical PSP amplitude	*A* = 3.25 *mV*, *B* = 22 *mV*
Inverse of the dendritic conduction time	*a* = 100 *Hz*, *b* = 50 *Hz*
Intra-column coupling	*C* = 133.5 *C*1 = *C*, *C*2 = 0.8*C* *C*3 = 0.25*C*, *C*4 = 0.25*C*
Maximum average action potential density	*e*_0_ = 2.5 *Hz*
Steepness of the response function	*r* = 0.56 mV^−1^
PSP for a 50% firing rate	*ν*_0_ = 6.0 *mV*
Constant external input	$\bar{p}$ = 155.0 *Hz*
Amplitude of the periodic driving	*δ* = 65.0 *Hz*
Frequency of the periodic driving	*f* = 8.5 *Hz*

**Figure 1:**
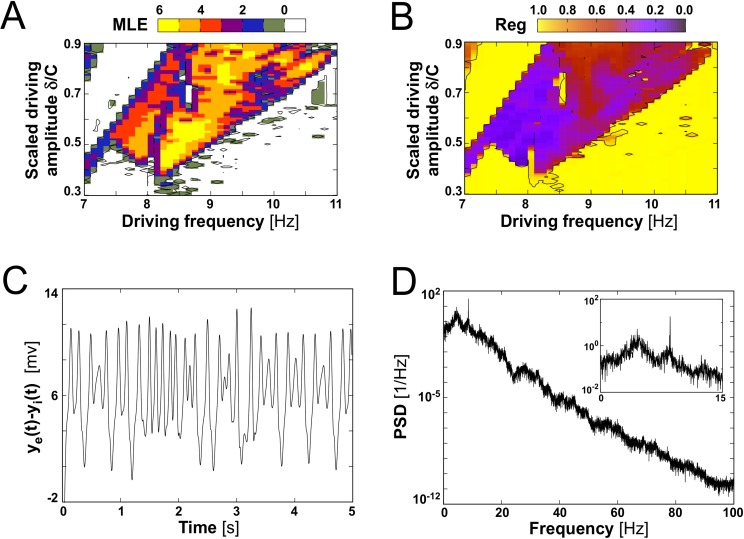
Chaotic behavior of an isolated, periodically driven cortical column. (A) Maximal Lyapunov Exponent (MLE) for different driving amplitudes and frequencies. Parameter values are those given in Table 1. (B) Regularity parameter obtained in the same conditions as in panel A. (C) Example of a chaotic signal obtained for a driving frequency *f* = 8.5 *Hz* and scaled driving amplitude *δ*/*C* = 0.49 *Hz*. With these parameters MLE = 5.24 and *Reg* = 0.37. (D) Power spectrum of the signal shown in C. This complex spectrum shows a narrow peak at the driving frequency (see inset).

Chaotic dynamics appears in this system when adding a periodic spike density, *δ*sin(2*πft*), to the constant external input, $\bar{p}$. The irregularity of this dynamics can be quantified by computing the Maximal Lyapunov Exponent (MLE) [74], as shown in Fig. 1A. That plot represents the MLE obtained for a periodically forced single cortical column when varying its driving amplitude *δ* and frequency *f*. A positive MLE corresponds to a chaotic dynamical evolution of the system. It is noticeable that many combinations of driving frequency and amplitude lead to chaos, and that a small domain characterized by negative MLE, with *f* ∼ 8.7 *Hz* and scaled driving amplitude *δ*/*C* around 0.7 *Hz*, appears inside the chaotic domain.

Calculating the MLE becomes computationally costly when dealing with a large number of columns. Thus, in the rest of the paper we will use a different measure of the regularity of the dynamics, given by the parameter *Reg*. This quantity is calculated using the second maximum of the autocorrelation function for *y*_*e*_(*t*)−*y*_*i*_(*t*) signal (see Eqs. (9, 10, 11), as defined in Eq. (17) of the Materials and Methods section. Fig. 1B shows the values taken by this parameter as a function of *δ* and *f*. The regions characterized by large positive (negative) values of the MLE in Fig. 1A correspond to regions with low (high) value of *Reg* in Fig. 1B. An example of chaotic evolution for driving frequency *f* = 8.5 *Hz* and scaled driving amplitude *δ*/*C* = 0.49 *Hz* is illustrated by the signal dynamics and its corresponding power spectrum density (PSD) in Fig. 1C and D, respectively. These results show that the response of a node to a simple periodic driving may be very complex in both the time and frequency domains. The parameter *Reg* will be used hereafter to quantify the regularity of the signals that characterize the network dynamics.

### Network activity

**Figure 2:**
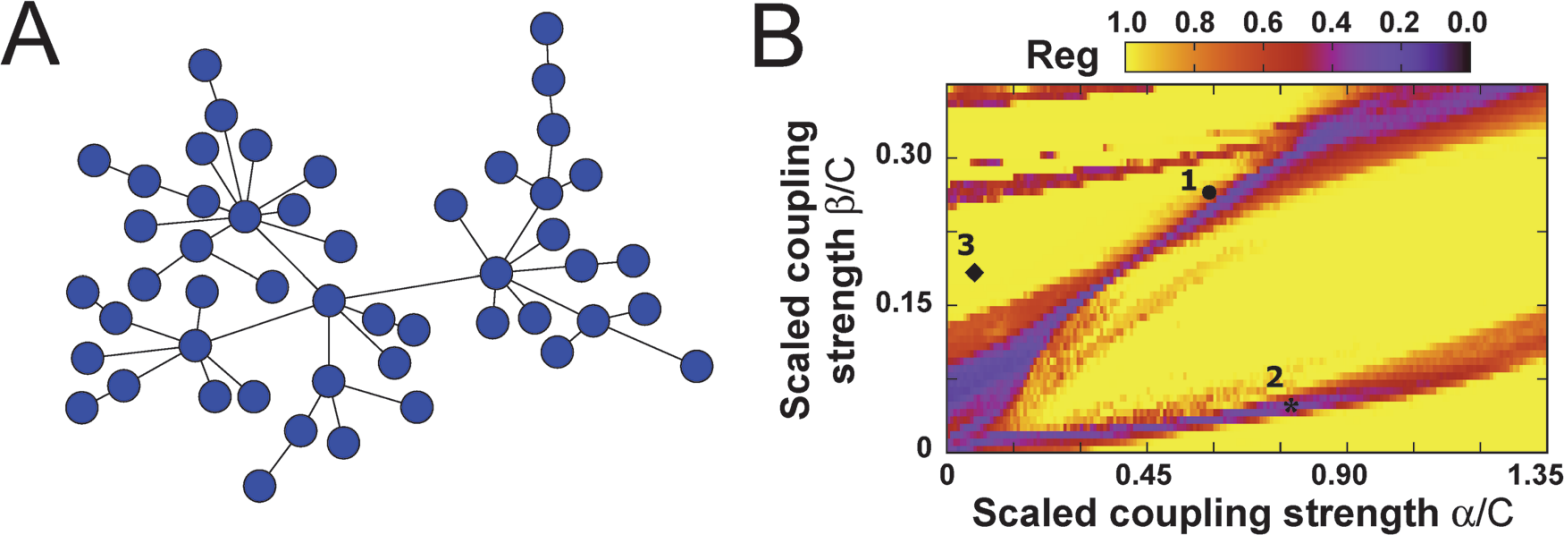
Regularity of the coupled system. (A) Topology of the network of cortical macrocolumns studied below. The network is constructed using the Barabási-Albert algorithm with *m*_0_ = 1 initial nodes (see Materials and Methods for details). (B) Regularity parameter averaged over 1 network of 50 coupled cortical columns (see panel (A)), for 20 different realizations of the initial conditions and for varying excitatory and inhibitory coupling intensities. Darker regions indicate less regular dynamics (chaotic), whereas lighter regions indicate more regular dynamics. Annotations in the plot indicate parameter values that will be studied later in Figs. 3, 4A, 4C, 6A and 6B.

Our study is centered in a 50 node network with inhomogeneous connectivity, represented in Fig. 2A. The nodes’ degree distribution in such network follows a power-law (but see also results for other topologies in Supporting Information Fig. S2). A full characterization of the network and the coupling scheme is presented in the Materials and Methods section, together with a complete mathematical description of the model. The network study was conducted using a fixed set of driving parameters, $\bar{p}$, *δ* and *f*, which for isolated nodes produced irregular dynamical evolutions (see Table 1 and Fig. 1C). All nodes of the network are identical and receive the same external input. The input contribution resulting from the neighbors is weighted for each node in such a way that every node receives an input spike density within the same total bounded range (see Materials and Methods Eqs.6–7) for more details).

The inter-columnar coupling intensities are determined by parameters *α* and *β* for the excitatory and inhibitory coupling, respectively (see Eqs.(6, 7)). We calculated the regularity averaged across the fifty nodes of a single network and over twenty different random realizations of the initial conditions. Fig. 2B shows the map of the average regularity for the coupled nodes as a function of the two coupling intensities. This figure shows that certain regions of parameter space exhibit a low level of regularity. In particular, for a fixed value of the excitatory coupling *α*, the dynamics becomes irregular for both sufficiently large and sufficiently small values of the inhibitory coupling *β*. The distribution of these irregular regions, in terms of *α* and *β*, depends on the other parameters of the system. For some *α*-*β* combinations (shown in more detail below) periodic, quasi-periodic and chaotic nodes coexist in a single network, even though all nodes receive the same driving. For other *α*-*β* combinations, the whole network exhibits either highly regular or highly irregular dynamics.

### Correlation between pairs of nodes

**Figure 3:**
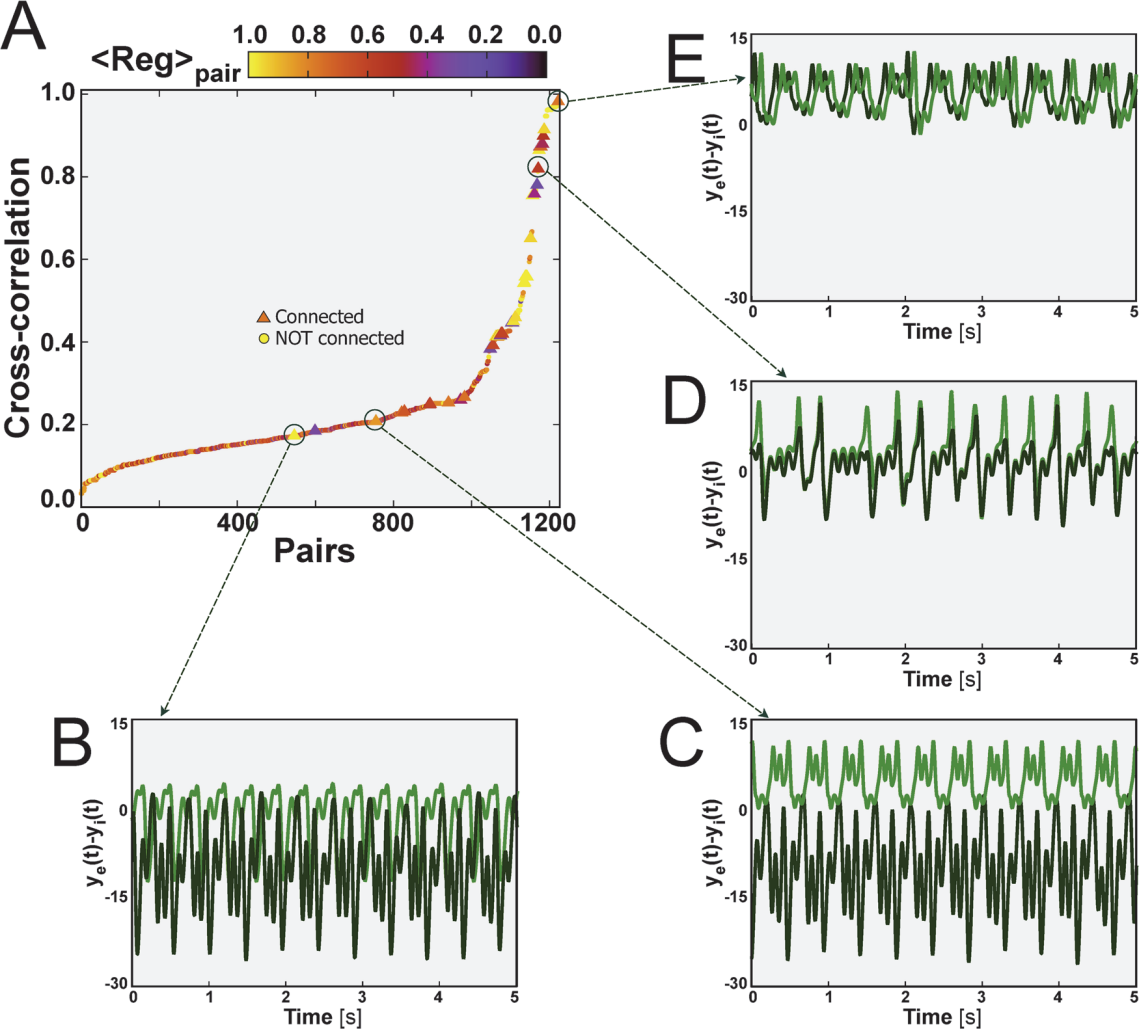
Node-pair correlation and dynamical clustering. (A) Maximal *C*_*max*_(*τ*) (y axis) and average regularity (color coded) between pairs of cortical columns for scaled *α*/*C* = 0.56 and *β*/*C* = 0.26 (point 1 in Fig. 2B). Connected (not connected) pairs of nodes are shown as triangles (circles). The dynamical evolution of selected node pairs is shown in panels B-E. In (E) the two nodes are synchronized at zero lag, and one of the time traces has been shifted horizontally for clarity.

The inter-columnar coupling intensities determine not only the regularity of the dynamics of the individual nodes, but also their synchronization capacity. In particular, for fixed *α*-*β* values, different degrees of synchronization appear between different pairs of nodes of the network. Fig. 3A plots the maximal cross-correlation values, *C*_*max*_(*τ*), for all pairs of nodes *i*,*j*, ordered for increasing correlation, for scaled *α*/*C* = 0.56 and *β*/*C* = 0.26 (labeled as point 1 in Fig. 2B). Directly connected pairs, represented by triangles in Fig. 3A, have values of *C*_*max*_(*τ*) in the range 0.2–1, and lie mostly above *C*_*max*_(*τ*) = 0.4, indicating that the activity of direct neighbors is highly correlated. For pairs of nodes that are not connected directly (shown as circles in Fig. 3A) the cross correlation is spread across the entire range 0–1. In order to relate the amount of correlation between node pairs with the regularity of their dynamics, Fig. 3A shows in color coding the regularity parameter described above (averaged over the two nodes in the pair). For the set of parameters considered in Fig. 3A, the network-averaged regularity is *Reg* ≈ 0.6 (see point 1 in Fig. 2B). In that case there are node pairs evolving in a regular way (lighter symbols, see e.g. Fig. 3B,C) or in a more irregular manner (darker symbols, see e.g. Fig. 3D,E), although there is no clear association between regularity and correlation level.

As described in the Materials and Methods section, in the neural mass model used here the activity of each node is described by the excitatory (EPSP) and inhibitory (IPSP) inputs to the pyramidal population (*y*_*e*_(*t*) and *y*_*i*_(*t*), respectively). Hence, each node (representing a cortical column) can be considered inhibitory if ⟨*y*_*e*_(*t*)−*y*_*i*_(*t*)⟩ < 0, and excitatory if ⟨*y*_*e*_(*t*)−*y*_*i*_(*t*)⟩ ≥ 0, where ⟨⋯⟩ denotes temporal average. Fig. 3B shows the dynamics of the total input signal *y*(*t*) = *y*_*e*_(*t*)−*y*_*i*_(*t*) for a pair of directly connected nodes characterized by high regularity and low correlated activity. In this case, the activity of both nodes is dominantly inhibitory. In contrast, the pair of nodes illustrated in Fig. 3C (which are also directly connected and have low correlation) have opposite activity, with one node being purely excitatory (light green curve), while the other is mainly inhibitory (dark green curve). The separation of the activities of the two nodes in two dominant exclusive types (one excitatory and one inhibitory) is an emergent feature that we have termed *segregation*, and which is discussed in detail in the next section. Fig. 3D shows an example of highly correlated activity and low regularity of two directly connected cortical columns. Note that in this case there is no dominant activity (neither excitatory nor inhibitory) of any of the two nodes. Finally, Fig. 3E shows very highly correlated activity for a pair nodes that are not directly connected. In this case the correlation is so strong that the two nodes exhibit exactly the same dynamics, with zero-lag synchronization (one of the time traces as been slightly shifted horizontally so that the two series can be discerned). This unconnected pair is coupled indirectly through a third node, a feature that can give rise to zero-lag synchronization [76].

### Excitatory/inhibitory segregation

**Figure 4:**
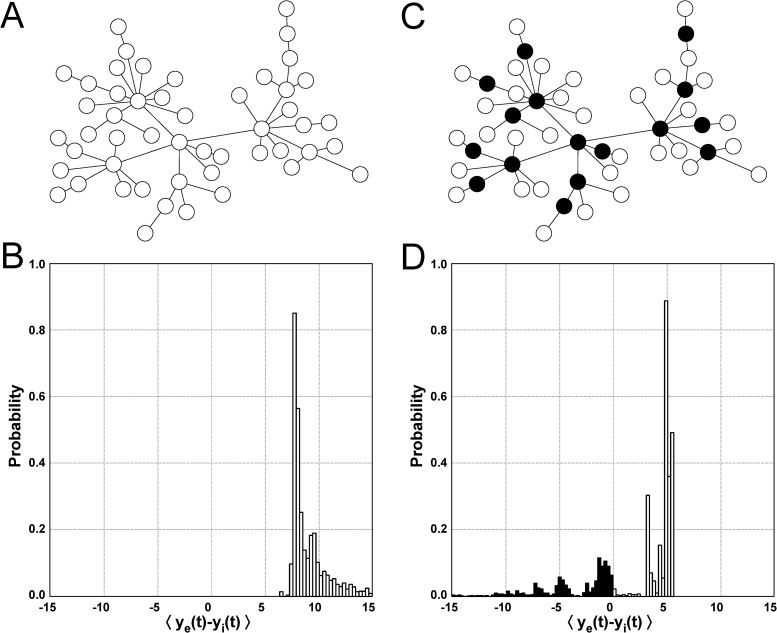
Excitatory/inhibitory segregation of cortical columns. (A) Example of a scale-free network characterized by all nodes exhibiting a dominant excitatory dynamics, illustrated by empty circles. Here *α*/*C* = 0.790 and *β*/*C* = 0.037 (Fig. 2B, point 2). (B) Average distribution of the activity ⟨*y*_*e*_(*t*)−*y*_*i*_(*t*)⟩, obtained from the analysis of 50 different scale-free architectures using different random seeds for the same *α* and *β* parameters as in panel A. The extreme values in the tail of the distribution are not represented. (C) The same scale-free network of panel A but now for the case of a mostly inhibitory intercolumnar coupling, with *β*/*C* = 0.190 compared to *α*/*C* = 0.075 (Fig. 2B, point 3). Here full circles represent inhibitory nodes, and empty circles denote again excitatory nodes. (D) Average distribution of ⟨*y*_*e*_(*t*)−*y*_*i*_(*t*)⟩ corresponding to panel C.

The results from Fig. 3 show that coupled neural masses can exhibit a regime of partial synchronization in which the activity of the nodes is segregated between mostly excitatory and mostly inhibitory activity (e.g. Fig. 3C). We now ask how widespread this segregated dynamics is, and how it depends on the values of the inter-columnar coupling strengths *α* and *β*. For scaled *α*/*C* = 0.790 and *β*/*C* = 0.037 (point 2 in Fig. 2B), for instance, the coupled nodes show on average a low level of regularity. Despite the irregularity of the dynamics this is a case of overwhelming excitatory behavior and no segregation is observed (Fig. 4A). In order to obtain a reliable statistics independent of the initial conditions, we run a set of 50 simulations, each one with a different network, constructed as described in the Materials and Methods section, with the same topological parameters and different random seeds. In this case the activity *y* = ⟨*y*_*e*_(*t*)−*y*_*i*_(*t*)⟩ of all nodes is positive (Fig. 4B). Note that *α* ≫ *β* in this case, which corresponds to the inter-columnar coupling being dominated by excitation. In the opposite case (e.g. scaled *α*/*C* = 0.075 and *β*/*C* = 0.190, point 3 in Fig. 2B), the dynamics of the nodes is more regular and segregation arises, as illustrated by Fig. 4C. In this case a large fraction of the nodes maintain a dominantly excitatory activity, but approximately one fifth of the nodes exhibit a dominantly inhibitory dynamics (Fig. 4D). Visual inspection of Fig. 4C seems to indicate that the connectivity hubs of the network are preferentially inhibitory, which will be quantitatively tested below.

**Figure 5:**
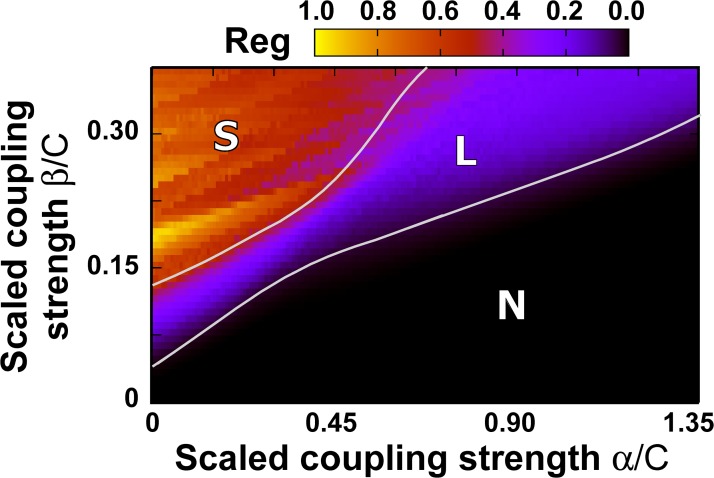
Excitatory-Inhibitory Segregation index (EIS). The EIS index quantifies the relative distribution of excitatory- and inhibitory-dominated dynamics. Three different domains exist, labeled by ‘N’ (no segregation), ‘L’ (low segregation) and ‘S’(high segregation).

In order to establish the robustness of the segregation, we now compute the excitation/inhibition segregation index (EIS), as defined in the section Materials and Methods, in the parameter space defined by the coupling parameters *α* and *β*. This index compares the excitatory and inhibitory sides of the distribution of ⟨*y*_*e*_(*t*)−*y*_*i*_(*t*)⟩ (as shown in plots B and D of Fig. 4). A value *EIS* = 0 means that there is no separation between excitation and inhibition (all nodes are either purely excitatory or purely inhibitory), whereas large values of *EIS* mean that the number of nodes is evenly distributed in each side of the histogram, with a large difference of the ⟨*y*_*e*_(*t*)−*y*_*i*_(*t*)⟩ value between excitatory and inhibitory nodes. Fig. 5 shows the distribution of EIS for the region of *α*-*β* parameter space studied so far. We observe that the dynamics is roughly separated in three domains. A domain characterized by no segregation, labeled as ‘N’ in the plot, corresponds to a dominantly excitatory input. An intermediate domain, labeled ‘L’, is characterized by a low level of segregation. Finally, if *β* is large enough and *α* is small enough, a regime of high segregation, labeled ‘S’, arises. Note that the transitions between the three domains are rather sharp.

**Figure 6:**
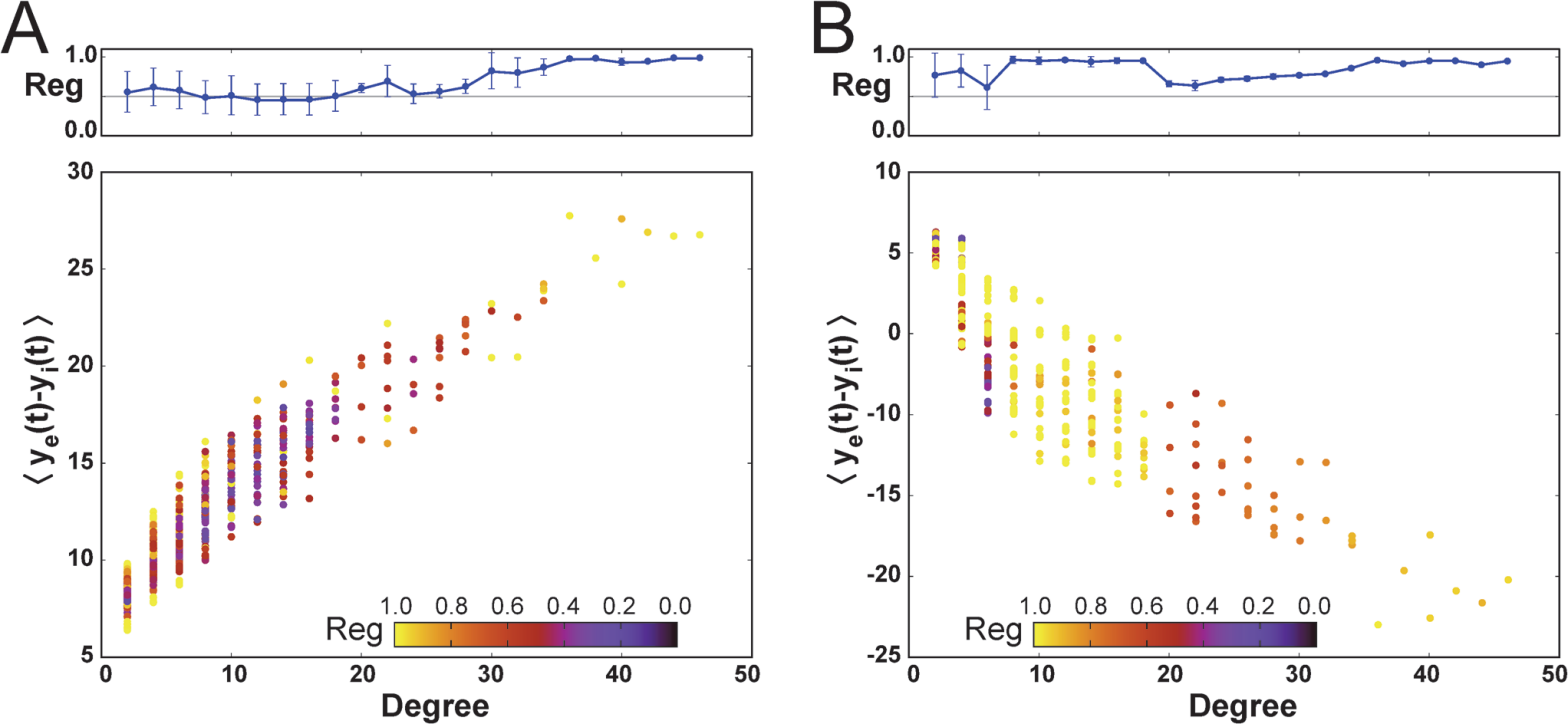
Topological organization of the excitation-inhibition segregation. Average activity of all nodes of the networks as a function of their degree, corresponding to the two cases analyzed in Figs. 4A and 4C, respectively. Color coding denotes the regularity for each node in the networks, and upper panels show the average regularity in the nodes’ dynamics as well as its standard deviation.

In order to determine the influence of the network characteristics on the dynamics of the nodes, we examined the relationship between the number of connections of each node (degree), its average activity ⟨*y*_*e*_(*t*)−*y*_*i*_(*t*)⟩, and its regularity. Fig. 6 shows the results for the *α*-*β* combinations used in Fig. 4. The first feature that is evident from those plots is that the average activity of the nodes is strongly correlated with their degree, both when the inter-columnar coupling is mainly excitatory (Fig. 6A) and when it is mostly inhibitory (Fig. 6B). This can be understood from the fact that hubs receive input from a large number of nodes, and if this input is mostly excitatory (as in Fig. 6A) the resulting activity of the node will be strongly excitatory, more so than nodes with a small number of inputs. Conversely, if the coupling is mainly inhibitory (as in Fig. 6B), the activity of the hubs will be dominantly inhibitory, while the lowly connected nodes will receive a weaker inhibitory input. In this case, however, and in contrast with the situation of Fig. 6A, the weak inhibitory input received by the low-degree nodes will be counterbalanced by an external excitatory input ($\bar{p}$ as defined in the Materials and Methods section), which acts upon all nodes of the network. For appropriate parameter values this external input dominates over the input coming from the neighboring nodes, resulting in an average activity that is predominantly excitatory for these low-degree nodes, while the highly connected nodes behave in an inhibitory manner. This leads to segregation between inhibitory and excitatory nodes, as described above and shown in Fig. 4 and 5, with the inhibitory role taken over by the network hubs.

There is also an important difference in the distribution of regularity of the nodes, depending on the segregation exhibited by the network. In the absence of segregation (Fig. 6A), the nodes of intermediate degree exhibit a broad range of regularity, from very irregular to very regular modes. In contrast, in the presence of segregation (Fig. 6B) the intermediate-degree nodes display highly regular dynamics. This is due to the fact that in the latter case the dominant activity is inhibitory, and inhibition reduces irregularity and favors synchronization. A common feature of the networks, irrespective of the segregation level, is that low-degree nodes exhibit a large diversity of regularities. This happens because the regularity of the dynamics of a node will depend on the degree of the neighbors that are connected to it, and thus the low-degree nodes might be highly influenced by other low-degree nodes (given that the coupling is scaled by the inverse of the product of the degrees of the two connected nodes, see Materials and Methods section) or weakly influenced by high-degree columns.

**Figure 7:**
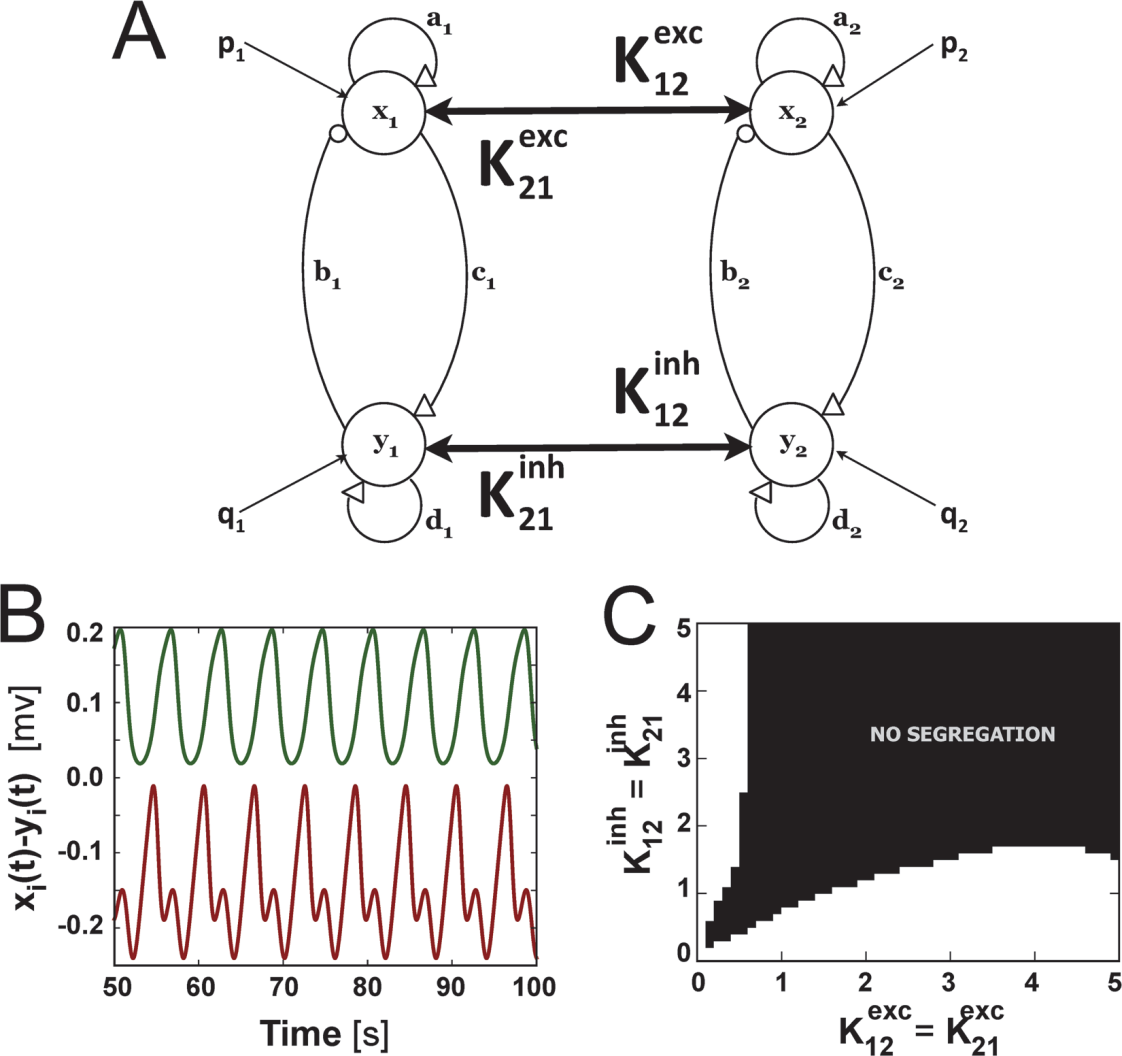
Segregation of two coupled Wilson-Cowan oscillators. (A) Coupling scheme between the two oscillators. (B) Time traces of the subtracted signal *x*_*i*_−*y*_*i*_ for the two segregated oscillators, in an excitatory-dominated coupling scheme (*K*_*exc*_ = 1, *K*_*inh*_ = 0.1). (C) Resulting segregation map for different excitatory and inhibitory coupling strengths.

As a final remark, the emergence of segregation of excitation and inhibition is also present in other mesoscopic descriptions of the neuronal activity, showing the possible universality of the phenomenon. To proof so, we studied the dynamics of two coupled Wilson-Cowan oscillators [77, 78] (see Fig. 7A), which showed different levels of segregation depending on the excitatory and the inhibitory coupling strengths $K_{ij}^{exc},\;K_{ij}^{inh}$ (see details of the model in the Materials and Methods section). This is shown in Fig. 1B in terms of *x*(*t*)−*y*(*t*), which resembles the observable typically used in the Jansen and Rit model. Besides, segregation occurs when the excitatory and the inhibitory blocks of each oscillator receive a different level of external stimulus, that is, *p*_1_ ≠ *p*_2_ as well as *q*_1_ ≠ *q*_2_ (being *p*_*i*_ ≠ *q*_*i*_).

In Fig. 7C we have calculated the average excitability as ⟨*x*−*y*⟩_*t*_ and plotted the $K_{ij}^{exc},\;K_{ij}^{inh}$ values for which ⟨*x*_1_−*y*_1_⟩_*t*_ and ⟨*x*_2_−*y*_2_⟩_*t*_ signals remain segregated. For the case of Wilson-Cowan oscillators segregation might be excitatory or inhibitory dominated, depending on which coupling term ($K_{ij}^{exc}$ or $K_{ij}^{inh}$) is higher (as shown in Fig. 7C white regions).

We argue, then, that segregation may arise thanks to some minimal ingredients: the presence of excitatory and inhibitory blocks, the possibility of coupling such blocks through excitatory and inhibitory connections and an adequate range of coupling strengths.

## Discussion

Our model makes several assumptions. First, we consider that the cortical macrocolumns are subject to a periodic excitatory input that may have different origins. It may represent, for instance, the input activity from a nearby cortical column [50], a sensory input reflecting periodic stimulation [74] or the afferent input from a sub-cortical structure (such as the thalamus) [79]. As a result of this periodic driving we observe chaotic dynamics in single columns, in accordance with previous studies [74]. This irregular regime appears in broad regions of the parameter space defined by the frequency and amplitude of the input signal (Fig. 1A,B).

A second assumption of the model is the heterogeneous nature of the connectivity between coupled neuronal populations. This implies that a small number of cortical columns are more strongly connected than the majority of the nodes in the neural mass network. Such heterogeneous connectivity profile is supported by experimental observations [59, 68–71]. A third assumption of the model is that the coupling between the cortical columns can be excitatory or inhibitory. We can expect this to be the case if the networks that we consider describe local brain areas communicating via short-range connections.

We choose our network of cortical columns to operate in the alpha band. Our analysis of the average regularity of the coupled nodes as a function of the inter-columnar excitatory and inhibitory coupling intensities reveals that the network can include nodes in different states (periodic, quasi-periodic, and chaotic). These results are also in agreement with the description of chaotic dynamics at a cellular level following periodic inhibitory inputs [80, 81]. The specific dynamics is controlled by the nature of the inter-columnar coupling, reflecting the dependence of the oscillations on the excitatory and inhibitory interactions at the mesoscopic scale. On this basis we can suggest that a sudden alteration of the inter-columnar coupling intensities (e.g. in an epileptic state) is associated with the modification of the regularity of the mesoscopic activity, and that with the capacity of information processing by the network.

One of the main features of the networks studied here is that even when the nodes are identical and receive a common driving input, their activity can be segregated in two different modes, being predominantly excitatory or predominantly inhibitory. This segregation results from the combined effect of the heterogeneous network connectivity, the specific excitatory-inhibitory couplings, and the external excitatory input to which all network nodes are subject. However, if no external input is fed into the columns segregation also emerges, though in lower levels, as shown in small motifs of connected cortical modules [82]. The same phenomenon was observed in Wilson-Cowan oscillators [77] (see Fig. 7). Thus, external stimuli enrich the dynamics, in the form of chaotic-like time evolutions, and also enhances segregation, but it is not crucial for its emergence.

At the microscopic level, segregation of excitatory and inhibitory behavior is well known [3, 15–18, 20, 21]. The potential contribution of this phenomenon to a broader information processing capacity of mesoscopic brain networks results from the fact that segregated nodes explore dynamically a wider range of activity states without (necessarily) losing their capacity to evolve irregularly in a synchronized way with the rest of the network elements. Our study shows that mesoscopic segregation arises when inter-column couplings are predominantly inhibitory. As mentioned above, here we have concentrated on a network architecture with inhomogeneous degree, which is one of the possible anatomical and functional based architectures in the brain [59]. However, we thoroughly studied different network architectures ranging from regular rings to all-to-all topologies (results not shown for the latter). We have analyzed scale-free arrangements other than the one presented in Fig. 2A. Fig. S1 in the Supporting Information section shows how segregation is distributed in scale-free networks for increasing clustering constructed using *m*_0_ = 10 (Fig. S1A) and *m*_0_ = 20 (Fig. S1B) initial nodes. In turn, Fig. S2 shows how segregation is distributed in regular ring networks (panels A and B), in small-world networks (panels C and D) and in random networks (panels E and F). All these networks were constructed using the same number of nodes and the same number of realizations (initial conditions) as the networks studied above (see details in the Materials and Methods section). As it can be seen from these results, the emergence of excitatory-inhibitory segregation discussed here was typically found for complex topologies in contrast with regular topologies which do not exhibit segregated dynamics (see *EIS* map in Fig. S2A,B). The emergence of segregation is, thus, highly dependent on the topological features of the networks.

In a real brain, driving inputs are not as simple as the periodic input considered here, and produce highly irregular average dynamics at the mesoscopic level of cortical macrocolumns. This irregular behavior can be expected to have a higher information content (e.g. in terms of Shannon/transfer entropy) than much simpler and regular signals. The normal functioning state of the brain, however, requires a certain degree of synchrony [83]. A dysfunction in that synchrony leads often to aberrant behaviors and neurological diseases [84, 85]. For healthy working brains, it is therefore important to achieve a state in which coordinated irregular dynamics works to process information efficiently but non-trivially [86, 87]. In that way, the right amount of synchronization of the brain activity permits cooperative processing of information, thus increasing the computational power of the system [83]. The results presented here suggest that organization of excitation and inhibition at the mesoscopic level might contribute to this cooperative information processing.

## Materials and Methods

### Jansen and Rit Model

We study a system of coupled cortical macrocolumns described by an extension of the model developed by Jansen et al [35]. This model accounts for the dynamics of a cortical column by using a mean field approximation to represent the average activity of three interacting cortical populations: excitatory and inhibitory interneurons and pyramidal cells. The main pyramidal population excites the two interneuronal populations in a feedforward manner, while the excitatory (inhibitory) interneurons feed back into the pyramidal population in an excitatory (inhibitory) manner. The dynamical evolution of these three populations arises from two different transformations. First, each population transforms the total average density of action potentials that reach their synapses, ∑_*m*_ *p*_*m*_(*t*), into an average postsynaptic (excitatory or inhibitory) membrane potential *y*_*n*_(*t*). A linear convolution implements this transformation in terms of the kernel

$$h_{e}\left(t\right)=\left\{\begin{array}{cc} Aate^{-at} & \text{if}\;t\geq 0\\ 0 & \text{if}\;t<0, \end{array}\right.$$ (1)

for the excitatory couplings and

$$h_{i}\left(t\right)=\left\{\begin{array}{cc} Bbte^{-bt} & \text{if}\;t\geq 0\\ 0 & \text{if}\;t<0, \end{array}\right.$$ (2)

for the inhibitory couplings. *A* and *B* are related with the maximum height of the excitatory and inhibitory postsynaptic potentials (EPSP and IPSP, respectively), whereas *a* and *b* represent the inverse of the membrane time constants and the dendritic delays.

The second dynamical transformation is the conversion of the net average membrane potential into an average spike density. This conversion is done at the somas of neurons in the population, and is described mathematically by a sigmoidal function defined as:

$$S\left(m\left(t\right)\right)=\frac{2e_{0}}{1+e^{r(\nu_{0}-m\left(t\right))}}.$$ (3)

Here *e*_0_ determines the maximum firing rate of the neural population, *ν*_0_ sets the net PSP for which a 50% firing rate is achieved, *r* is the steepness of the sigmoidal transformation, and *m*(*t*) corresponds to the net PSP input into the considered population (see Table 1 for parameter values). The average density of action potentials produced by the presynaptic population and affecting the postsynaptic population turns out to be *p*_*m*_(*t*) = *λS*(*m*(*t*)), where *λ* weights the coupling between two population contacts. This coupling constant quantifies the efficiency of the synaptic contacts. The intra-columnar connectivity constants values are defined in terms of *C*_*p*_, with *p* = 1,...,4, as described by Jansen et al. [35]. Here *C* = 133.5.

In our model the different cortical columns interact with each other through both excitatory and inhibitory connections. The pyramidal-pyramidal excitatory couplings and the inhibitory interneuron-pyramidal inhibitory couplings mimic short range inputs from nearby columns. These inter-column interactions correspond to incoming pulse densities *p*_*α*_(*t*), for the excitatory input into the pyramidal population, and *p*_*β*_(*t*) for the inhibitory input into the pyramidal population, respectively. We chose this contact arrangement because pyramidal cells are widely regarded as the responsible for non local connectivity.

Besides the intra-columnar inputs, the pyramidal population receives a constant incoming pulse train of density $\bar{p}$ and a periodic driving *p*_*T*_(*t*) from external sources (e.g. subcortical structure such as the thalamus). The excitatory pulse densities reaching a cortical column *i* can be written as

$$\bar{p}\;=\;155.0,$$ (4)

$$p_{T}^{i}(t)\;=\;\delta \sin(2\pi ft),$$ (5)

$$p_{\alpha }^{i}(t)\;=\;\alpha \sum_{\begin{array}{c} j=1\\ j\neq i \end{array}}^{K}\frac{1}{\sqrt{N_{i}N_{j}}}\;S(y_{1}^{j}(t)-y_{2}^{j}(t)),$$ (6)

and the inhibitory pulse densities are

$$p_{\beta }^{i}(t)=\beta \sum_{\begin{array}{c} j=1\\ j\neq i \end{array}}^{K}\frac{1}{\sqrt{N_{i}N_{j}}}\;S(C_{3}y_{0}^{j}(t)).$$ (7)

Here *N*_*i*_ corresponds to the in-degree (number of connections entering the node) of the receiver node, *N*_*j*_ is the out-degree (number of connections leaving the node) of the emitter nodes and *K* is node’s *i* number of neighbors. All the couplings between neighbor nodes in the network are bidirectional. The transformation described by Equations (1) and (2) can be introduced using a differential operator $L(y_{n}^{i}(t);a)$ (*n* = 0,1) describing the excitatory integration of the average density of action potentials reaching the population:

$$L\left(y_{n}^{i}\left(t\right);a\right)=\frac{d^{2}y_{n}^{i}\left(t\right)}{dt^{2}}+2a\frac{dy_{n}^{i}\left(t\right)}{dt}+a^{2}y_{n}^{i}\left(t\right)=Aa\left[\sum_{m}p_{m}^{i}\left(t\right)\right],$$ (8)

Similarly we can define $L(y_{n}^{i}(t);b)$ (*n* = 2) to describe the inhibitory integration of the average density of action potentials.

We are centered in a network formed by a set of *N* = 50 cortical columns with a heterogeneous connectivity, constructed using a preferential attachment rule [88, 89]. The equations describing a node *i* (with *i* = 1,...*N*) are as follows:

$$L(y_{0}^{i}(t);a)\;=\;Aa\{S(y_{1}^{i}(t)-y_{2}^{i}(t))\}$$ (9)

$$\begin{array}{c} L(y_{1}^{i}(t);a)\;=\;Aa\{\bar{p}+C_{2}S(C_{1}y_{0}^{i}(t))+\delta \sin(2\pi ft)\\ +\;\alpha \sum_{\begin{array}{c} j=1 \end{array}}^{K}\frac{1}{\sqrt{N_{i}N_{j}}}S(y_{1}^{j}(t)-y_{2}^{j}(t))\}\;j\neq i \end{array}$$ (10)

$$\begin{array}{c} L(y_{2}^{i}(t);b)\;=\;Bb\{C_{4}S(C_{3}y_{0}^{i}(t))\\ +\;\beta \overset{K}{\underset{\begin{array}{c} j=1 \end{array}}{\sum }}\frac{1}{\sqrt{N_{i}N_{j}}}S(C_{3}y_{0}^{j}(t))\}\;j\neq i \end{array}.$$ (11)

The parameter values used in this study are depicted in Table 1. The dynamical variables $y_{0,1,2}^{i}$ represent, for cortical column *i*, the excitatory postsynaptic potential input into the interneuron population, the excitatory postsynaptic potential input into the pyramidal population (*y*_*e*_(*t*) in the Results section), and the inhibitory postsynaptic potential input into the pyramidal population (*y*_*i*_(*t*) in the Results section), respectively. The subtraction between the postsynaptic potentials of the excitatory and inhibitory inputs afferencies to the pyramidal population ($y_{1}^{i}(t)-y_{2}^{i}(t)$ or $y_{e}^{i}(t)-y_{i}^{i}(t)$) defines the outcome that we analyze, and its dynamical evolution can be related with EEG or MEG signals.

### Wilson-Cowan model

We have also used the Wilson-Cowan model [77] to show segregation dynamics. It is one of the first mean field models, which describes the activity of two pools of interacting neurons—excitatory and inhibitory—by averaging out individual responses. The Wilson-Cowan model equations read as follows:

$$\dot{x}\;=-x+S(ax-by+p),$$ (12)

$$\dot{y}\;=\;-y+S(cx-dy+q),$$ (13)

S(v) = 1/(1 + exp(−v)), (14)

with *x* (*y*) being the average activity of the excitatory (inhibitory) populations. *a* and *d* are the self excitation parameters for *x* and *y* units, and *b* and *c* are the couplings from the inhibitory (excitatory) unit *y* (*x*) to the excitatory (inhibitory) unit *x* (*y*), respectively (see Fig. 7A for more details). *p* and *q* represent external stimuli impinging upon each population. Each unit, *x* and *y*, can be interpreted as the average activity of the excitatory and the inhibitory neuronal populations, respectively. Moreover, *S*(*v*) gives the proportion of excitatory (inhibitory) neurons receiving thresholded excitation per unit time. We chose as parameter values *a* = 16, *b* = 12, *c* = 16, *d* = −2 and varied the external inputs *p*−*q* to find values that allowed segregation in the coupled scenario.

The coupling is performed as shown in Fig. 7A. The equations for each oscillator read in this case:

$${\dot{x}}_{i}\;=\;-x_{i}+S(ax_{i}-by_{i}+p_{i})+K_{ij}^{exc}(x_{j}-x_{i}),$$ (15)

$${\dot{y}}_{i}\;=\;-y_{i}+S(cx_{i}-dy_{i}+q_{i})+K_{ij}^{inh}(y_{j}-y_{i}),$$ (16)

with *i*,*j* = 1,2. We have explored which values of the coupling strengths *K*_*ij*_ (with *K*_*ij*_ = *K*_*ji*_, either excitatory or inhibitory) allow the system to remain segregated for fixed values of external stimuli *p*_1_ = −3.5, *q*_2_ = −6.5, *p*_2_ = −1.0 and *q*_2_ = −4.0.

### Analysis

We characterize the regularity of the signals in terms of the autocorrelation function and the Maximal Lyapunov Exponent (MLE), the synchronization in terms of the cross-correlation, and the segregation by using the average activity $\langle y_{1}^{i}(t)-y_{2}^{i}(t)\rangle$ ($\langle y_{e}^{i}(t)-y_{i}^{i}(t)\rangle$). The degree of regularity of that activity was calculated by taking the average of the second absolute maxima of the autocorrelation function over all the nodes of the network:

$$Reg=\frac{1}{s}\frac{1}{N}\sum_{p=1}^{s}\sum_{q=1}^{N}h_{p,q}^{2nd}\left(\tau \right),$$ (17)

where *s* stands for the number of realizations for different initial conditions—50 in the case studied in Fig. 1B, 20 in the case studied in Fig. 2B and 10 for the subsequent regularity calculations—, *N* is the total number of cortical columns—50 for all cases—and $h_{p,q}^{2nd}(\tau )$ denotes the height of the second absolute peak of the autocorrelation function for each signal. This index provides us with a quantification of the periodicity of the signal. The power spectral density (PSD) measurements were computed using Welch’s average periodogram method. These calculations were performed using standard Python functions. The Lyapunov spectrum was calculated using a QR algorithm [90] but we just took its largest value or *Maximal Lyapunov Exponent* (MLE). The maximal cross-correlation for each pair of columns in the network, *C*_*max*_(*τ*), was computed using the maximum value of the cross-correlation function. We computed the excitatory-inhibitory segregation (EIS) index as *EIS* = ∣*CM*_*e*_ *A*_*e*_ *CM*_*i*_ *A*_*i*_∣, where *CM*_*e*_ (*CM*_*i*_) stands for the position of the center of mass of the excitatory—positive (inhibitory—negative) part of the activity distribution (Fig. 4B,D), and *A*_*e*_ (*A*_*i*_) is the corresponding area of the excitatory (inhibitory) distributions, respectively.

The integration of the model equations was performed using two methods. The Adams-Bashforth method was used for the MLE computation [90], with a time step of 1 ms and a total simulation time of 500 s, which was sufficient for the Lyapunov coefficients to converge. An initial time window of 100 s was omitted to avoid transients when computing the MLE. We calculated the Lyapunov spectrum for 50 different initial conditions. In the rest of the calculations we used Heun’s method to integrate the model equations [91]. Random number generation was implemented using standard GSL routines to set different initial conditions when performing the statistical analysis of data. Each simulation of the model had a time step of 1 ms and a total simulated time of *t* = 50 s. A period of 25 s was omitted to avoid transients.

Scale-free networks were constructed using the Barabási-Albert algorithm [89], which makes use of *m*_0_ initial nodes connected randomly to which other nodes are added gradually. These new nodes are connected to *m* ≤ *m*_0_ existing nodes with a probability that increases with the number of links of the already connected nodes. This procedure gives rise to networks with heavily connected nodes, and thus with a power-law distribution of degrees. In Figs.4 and 6 we performed the analysis for 50 scale-free networks with *m*_0_ = 1. In Fig. 5 we constructed 10 networks for each *α* and *β* pair. In the Supporting Information Fig. S1 we constructed 10 scale-free networks with *m*_0_ = 10 (Fig. S1A) and *m*_0_ = 20 (Fig. S1B), respectively, and, therefore, increasing clustering. Networks in Fig. S2 were constructed using the Watts-Strogatz algorithm with rewiring probabilities (*RPs*) of 0, 0.5 and 1 [92]. Each *RP* defines a different network: ring (*RP* = 0), small-world (*RP* = 0.5) and random (*RP* = 1.0). We constructed 10 networks for each *RP*. In all cases every network was simulated using 10 sets of different initial conditions for the nodes and were constructed using the NetworkX Python package.

## Supporting Information

S1 Fig Segregation and regularity for low and high clustering in scale-free networks. (A,top) Map showing the excitatory-inhibitory segregation index (EIS) for a scale-free network constructed using the Barabási-Albert algorithm with *m*_0_ = 10 (see Materials and Methods section for details). (A,bottom) Network realizations showing the excitatory/inhibitory character of each node (symbol type) and its regularity (color coding following the colorbar at the right). For low *α* and *β* values segregation does not occur (bottom-left network) and the nodes display irregular dynamics. When inhibition dominates over excitation (bottom-right network) the most connected nodes (hubs) tend to become inhibitory and the nodes become more regular. (B) Same as panel (a) but for larger clustering. Segregation here is higher than in networks with lower clustering (see colorbar ranges). There is a region of maximal segregation for dominating *β*.
(ZIP)

S2 Fig Segregation and regularity of regular, small-world and random networks. (A,B) Map showing the excitatory-inhibitory segregation (EIS) index for a ring shaped network (with 2 neighbors in (A) and 10 neighbors in (B)) and examples of the segregation and regularity of the networks. No segregation occurs for any *α* and *β* values explored. (C,D) Segregation appears for complex topologies such as small-world with a rewiring probability of 0.5. (E,F) Random networks also display segregation. In panels from (C) to (F) it is shown that segregation is strongly dependent on the number of neighbors of each node (see segregation color bar ranges).
(ZIP)

S3 Fig Segregation of two coupled Wilson-Cowan oscillators. (A) Coupling scheme between the two oscillators. (B) Resulting segregation map. (C) Time traces of the subtracted signal *x_i_*−*y_i_* for the two segregated oscillators, in an excitatory-dominated situation (*K_exc_* = 1, *K_inh_* = 0.1).
(ZIP)

S1 Text Wilson-Cowan model. Description of the Wilson-Cowan neural model and the emergence of segregation in two coupled Wilson-Cowan oscillators for increasing excitatory and inhibitory coupling strengths.
(PDF)
