# Supplementary material for: Mesoscopic Segregation of Excitation and Inhibition in a Brain Network Model
Source: PLoS Comput Biol. 2015 Feb 11;11(2):e1004007. doi: 10.1371/journal.pcbi.1004007 (PMC4324935; doi:10.1371/journal.pcbi.1004007)
Supplement: S1 Text — Description of the Wilson-Cowan neural model and the emergence of segregation in two coupled Wilson-Cowan oscillators for increasing excitatory and inhibitory coupling strengths. (PDF) [file pcbi.1004007.s004.pdf]

Supporting Information Text S1.

The Wilson-Cowan model equations read as follows:

$$\dot{x} = -x + S(ax - by + p), \quad (1)$$
$$\dot{y} = -y + S(cx - dy + q), \quad (2)$$
$$S(v) = 1/(1 + exp(-v)), \quad (3)$$

with $x(y)$ being the average activity of the excitatory (inhibitory) populations. We chose as parameter values $a = 16$, $b = 12$, $c = 16$, $d = -2$ and varied the external inputs $p - q$ (see more details in Wilson and Cowan, 1972). A system of two Wilson-Cowan oscillators shows segregation in very precise conditions. From the results shown in Supporting Information Fig. S3 we may say that segregation occurs when the excitatory and the inhibitory blocks among the oscillators receive a different level of external stimulus, that is, p_1 and p_2 are different as well as q_1 and q_2. Besides, $p_i \neq q_i$.

Due to their multistable character, already in the uncoupled situation, the oscillators show different levels of excitability. So segregated dynamics appears when there is a difference in p_i and q_i values. We have calculated the average excitability as $\langle x - y \rangle_t$, which resembles the definition typically used in the Jansen model. The coupling is performed as shown in Supporting Information Fig. S3 . The equations for each oscillator read in this case:

$$\dot{x}_i = -x_i + S(ax_i - by_i + p_i) + K_{ij}^{exc}(x_j - x_i), \quad (4)$$
$$\dot{y}_i = -y_i + S(cx_i - dy_i + q_i) + K_{ij}^{inh}(x_j - x_i), \quad (5)$$

with $i, j = 1, 2$. We have explored which values of the coupling strengths K_{ij} (with $K_{ij} = K_{ji}$, either excitatory or inhibitory) allow the system to remain segregated. In this case, segregation might be excitatory (inhibitory) dominated when the term K_{ij}^{exc} (K_{ij}^{inh}) is higher. Thus, when inhibition (excitation) dominates over excitation (inhibition) the two oscillators remain in different levels of excitability. Supporting Information Fig. S3B shows the coupling values for which segregation occurs. It has to be pointed out that inhibitory couplings have a drastic effect on the oscillatory regime in Wilson-Cowan oscillators, as they can bring the system to a fixed point.
